# The Presence of an Aberrant Right Hepatic Artery Did Not Influence Surgical and Oncological Outcomes After Pancreaticoduodenectomy: A Comprehensive Systematic Review and Meta-Analysis

**DOI:** 10.1007/s00268-023-07191-2

**Published:** 2023-10-10

**Authors:** Claudio Ricci, Laura Alberici, Margherita Minghetti, Carlo Ingaldi, Davide Giovanni Grego, Vincenzo D’Ambra, Ermenegilda De Dona, Riccardo Casadei

**Affiliations:** 1 Division of Pancreatic Surgery, IRCCS Azienda Ospedaliero-Universitaria Di Bologna, via Albertoni 15, 40138 Bologna, Italy; 2 Department of Internal Medicine and Surgery (DIMEC), Alma Mater Studiorum, University of Bologna, Bologna, Italy

## Abstract

**Background:**

The presence of an aberrant right hepatic artery (a-RHA) could influence the oncological and postoperative results after pancreaticoduodenectomy (PD).

**Methods:**

A systematic review and metanalysis were conducted, including all comparative studies having patients who underwent PD without (na-RHA) or with a-RHA. The results were reported as risk ratios (RRs), mean differences (MDs), or hazard ratios (HRs) with 95% confidence intervals (95 CI). The random effects model was used to calculate the effect sizes. The endpoints were distinguished as critical and important. Critical endpoints were: R1 resection, overall survival (OS), morbidity, mortality, and biliary fistula (BL). Important endpoints were: postoperative pancreatic fistula (POPF), delayed gastric emptying (DGE), post pancreatectomy hemorrhage (PPH), length of stay (LOS), and operative time (OT).

**Results:**

Considering the R1 rate no significant differences were observed between the two groups (RR 1.06; 0.89 to 1.27). The two groups have a similar OS (HR 0.95; 0.85 to 1.06). Postoperative morbidity and mortality were similar between the two groups, with a RR of 0.97 (0.88 to 1.06) and 0.81 (0.54 to 1.20), respectively. The biliary fistula rate was similar between the two groups (RR of 1.09; 0.72 to 1.66). No differences were observed for non-critical endpoints.

**Conclusion:**

The presence of a-RHA does not affect negatively the short-term and long-term clinical outcomes of PD.

## Introduction

An aberrant right hepatic artery (a-RHA) is frequently observed (15–35%) in patients undergoing pancreaticoduodenectomy (PD) [1]. This vascular abnormality could influence the oncological and postoperative results. On one side, the attempt to preserve the a-RHA could produce a lesser R0 rate on the superior mesenteric artery (SMA) margin, increasing the risk of local recurrence [2, 3]. Indeed, some authors [4] advocated the ligation or resection and reconstruction of a-RHA during PD [5]. On the other side, the aRHA ligation could produce reduced perfusion of biliary three and liver with an increased risk of biliary fistula, liver failure, or abscess. The ideal approach is the resection to obtain R0 resection and reconstruction to avoid the negative consequence in the postoperative course. However, a recent meta-analysis suggests that pancreatic surgeons rarely decide to resect the a-RHAs: Only 8% were sacrificed, and only 4% were resected and reconstructed [1]. The present study aimed to compare the oncological and postoperative results of patients with a-RHA who underwent PD. For this purpose, a systematic review and meta-analysis were carried out.

## Material and methods

The protocol was pre-registered in PROSPERO (code CRD42023426248). The systematic review was conducted in line with the Cochrane recommendations [6]. The manuscript was structured according to the PRISMA checklist (Preferred Reporting Items for Systematic Reviews and Meta-Analyses) [7].

### Eligibility criteria

The "Population-Intervention-Control-Outcomes-Studies" (PICOS) approach was used to establish the inclusion criteria [8]: The "Population" was represented by patients who underwent PD; the "Intervention" arm was represented by the patients with the aberrant right hepatic artery (a-RHA); "Control" arms included patients with normal hepatic artery or other anomalies that do not include an a-RHA (na-RHA); “Outcomes” were the radicality of resection (R0 vs. R1), postoperative complication including pancreatic fistula rate (CR-POPF), delayed gastric emptying (DGE), postpancreatectomy hemorrhage (PPH), overall survival (OS), disease-free survival (DFS), and operative time. It should be noted that the radicality of resection was calculated only for patients with malignant peri-ampullary cancer, excluding benign diseases. Only comparative, retrospective, or prospective studies were included.

### Information source, search, study selection, and data collection process

The systematic review was performed using PubMed, Scopus, and Web of Science. The string was developed in PubMed as follows: "("abnormalities"[MeSH Subheading] OR "abnormal*"[Text Word] OR "anomal*"[Text Word] OR "aberrant"[Text Word] OR "varia*"[Text Word] OR "anatomy varia*"[Text Word] OR "anatomical varia*"[Text Word]) AND ("Hepatic Artery"[MeSH Terms] OR "vascular*"[Text Word] OR "vessel*"[Text Word] OR "arter*"[Text Word] OR "Hepatic Artery"[Text Word] OR "replaced hepatic artery"[Text Word] OR "accessory hepatic artery"[Text Word] OR "right hepatic artery"[Text Word] OR "replaced right hepatic artery"[Text Word] OR "accessory right hepatic artery"[Text Word]) AND ("Pancreaticoduodenectomy"[MeSH Terms] OR "Pancreaticoduodenectomy"[Text Word] OR "Whipple"[Text Word] OR "Whipple procedure"[Text Word] OR "pancreatectomy"[Text Word] OR "pancreatic surgery"[Text Word] OR "pancreatic resection"[Text Word])." SR accelerator was used to translate the search string for Scopus and Web of Science [9]. The last search was carried out on April 30, 2023.

### Data items and risk of bias in individual study

For descriptive purposes, we extracted the following data: authors, year of publication, affiliation, country, classification of a-RHA, type of surgeons, frequency of not expendable RHA anomalies such as replaced RHA or replaced common hepatic artery (CHA) or celiac-mesenteric trunk (CMT). We also reported the number of resected a-RHA (with and without reconstruction and the frequency of a-RHA injured. Finally, also the rate of PDAC was extracted. The importance of outcomes was classified using the GRADE approach [10] (not important, important, critical). The R1 rate, overall survival (OS), postoperative morbidity, mortality, and biliary fistula were judged “critical.” The important but not critical endpoints were: clinically relevant POPF (CR-POPF), defined according to the updated ISGPF definition [11]; PPH and DGE according to the standardized definition [12, 13]; length of stay (LOS); and operative time (OP). The endpoints were described as frequencies and percentage or means and standard deviations (SD). The risk of bias within the individual studies was evaluated using the methodological index for non-randomized studies (MINORS) [14]. Two authors (LA and DGG) will extract the data using a dedicated spreadsheet. Any disagreement will be solved after a collegial discussion involving the first author (CR).

### Summary measurements and synthesis of the results

The mean and SD were obtained using a proper algorithm when the paper reported medians and interquartile or ranges [15, 16]. To obtain the hazard ratio from studies who not report this datum, we used a specific algorithm [17]^.^ We extracted the survival frequency from the Kaplan–Meier curves with dedicated open-source software (Engauge digitizer). The results were reported as risk ratios (RRs), mean differences (MDs), or hazard ratios (HRs) with 95% confidence intervals (95 CI). The Mantel–Haenszel (M-H) random effects model was used to calculate the effect sizes [18].

### Risk of bias across studies and meta-regression analysis

The heterogeneity was tested using *I*^2^ and Cochran's Q statistics [19]. Begg and the Egger tests [20] were employed to evaluate the presence of publication bias, and a *P*-value < 0.05 indicated a significant "small-study effect." The reasons for heterogeneity, when present (*I*^2^ > 50%), were investigated with a meta-regression analysis [21]. The covariates extracted for meta-regression were: country, standardized classification for a-RHA, type of surgeon, rate of PDAC in each group, and MINORS score. The presence of type I and type II error was also evaluated using trial sequential analysis [22]. As well known, in statistics, a false positive result is called a Type I error. Usually, the classical boundary to accept a result as “true” is prefixed at *p*-value < 0.05, which implies that the data only have a 5% probability of occurring as “false.” It should be noted that the sample size did not influence when a single study was considered. In a metanalysis, several studies are accrued in chronological order, and the type I error could be inflated. In other words, in a meta-analysis, the statistical significance could not be enough to reject the Type I error. The trial sequential analysis (TSA) checks the sample size to establish when the risk of Type I error can be excluded. In other words, TSA discloses when the false positive results are present also in the presence of a significant *p*-value [23]. On the contrary, false negative results can occur when nonsignificant *p*-values are observed, but this is related to the small sample size of the meta-analysis. The required sample size (RIS) is the number of patients sufficient to exclude both Type I and Type II errors. If RIS is equal or inferior to accrued sample size (ASS), the false negative and positive results were excluded.

## Results

### Studies selected

**Fig. 1:**
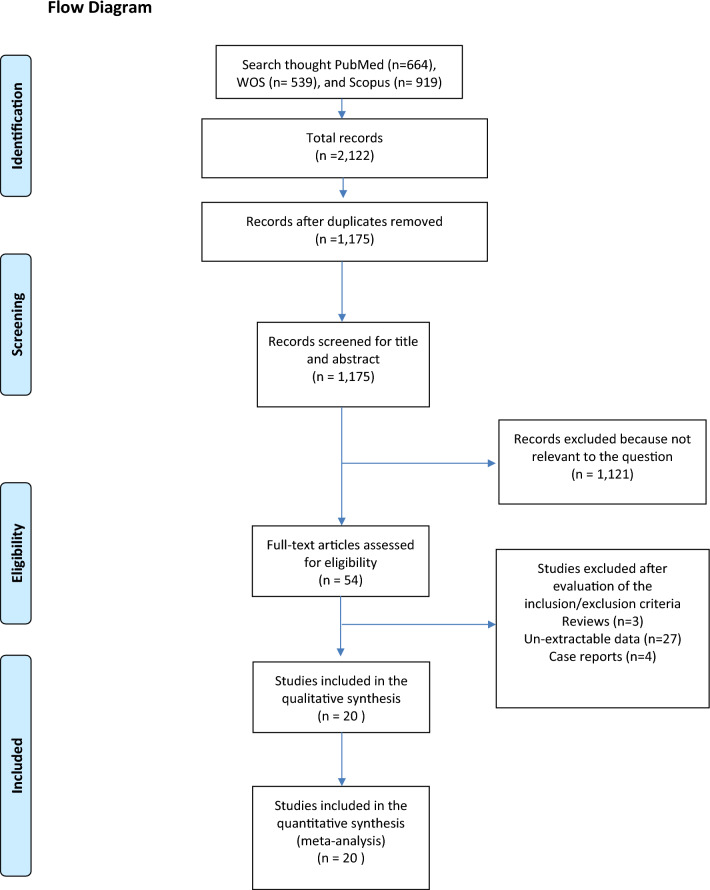
PRISMA Flowchart

The transparency of the selection is reported in Fig. 1. Twenty studies [3, 24–42] were eligible for the analysis.

### Study characteristics and risk of bias within studies

Table 1 reports the summary of included studies. Three (15%) studies were done in Eastern countries. Thirteen papers (65%) used a standardized classification of the a-RHAs. In nine (45%) studies, the surgeon was a dedicated pancreatic surgeon, while in five (25%) was an HPB surgeon. In the remaining five (25%) studies, the expertise of the surgical team was not declared. The overall rate of a-RHA was 20.5%, while the rate of replaced a-RHA, according to Michels classification, was 66.5%. The a-RHA was sacrificed in 6.5%, with a rate of injury of 2.5%. The rate of reconstruction with end-to-end anastomosis was low (1.8%). The overall rate of PDAC between the two groups was similar (RR 1.00; 0.97 to 1.02, 95 CI). The median MINORS score was 19.

**Table 1:** Characteristics of the 20 included studies

First Author/year	Affiliation/country	Classification of aberrant RHA	Type of surgeon	Rep RHA/CHA or CMT	RHA resected	RHA injured	RHA reconstructed	PDAC RR	MINORS
Jah et al. [24]	Department of Hepatobiliary and Transplant Surgery, Addenbrooke’s Hospital, Cambridge (UK)	Unstandardized	HPB	–	3/28 (10.7)	0/28 (0)	1/28 (3.6)	0.97 (0.55 to 1.70)	17
Lee et al. [25]	Department of Surgery, Ajou University School of Medicine, Suwon, (Republic of Korea)	Unstandardized	Unspecified	–	0/15 (0)	0/15 (0)	0/15 (0)	–	18
Eshuis et al. [26]	Department of Surgery, Academic Medical Centre, Amsterdam, the Netherlands	Michels/Hiatt	Unspecified	117	8/143 (5.6)	2/143 (1.4)	2/143 (1.4)	0.91 (0.72 to 1.15)	19
Perwaiz et al. [27]	Department of Surgical Gastroenterology, Sir Ganga Ram Hospital. New Delhi (India)	Michels	Unspecified	30	–	–	–	–	18
Turrini et al. [28]	Department of Surgery, Indiana University School of Medicine, Indianapolis, IN, (USA)	Michels	Unspecified	44	3/47 (6.4)	0/47 (0)	2/47 (4.2)	1.00 (0.95 to 1.05)	19
Sulpice et al. [29]	Chirurgie Hépatobiliaire et Digestive, Hôpital Pontchaillou, Rennes (France)	Michels	P	–	6/29 (20.7)	0/29 (0)	2/29 (6.9)	1.05 (0.77 to 1.45)	19
Kim et al. [30]	Departments of Surgical Oncology and Princess Margaret Cancer Center, Toronto, (Canada)	Michels	HPB	34	5/37 (13.5)	1/37 (2.7)	1/37 (2.7)	1.00 (0.96 to 1.04)	18
Rammohan et al. [31]	Surgical Gastroenterology and Liver Transplantation, College Hospital, Chennai, (India)	Michels	HPB	32	8/43 (18.6)	0/43 (0)	1/43 (2.3)	0.98 (0.43 to 2.25)	19
Dorado et al. [32]	HBP Surgery and Liver Transplantation Unit. Sevilla, (Spain)	Unstandardized	HPB	9	1/11 (9.1)	0/11 (0)	1/11 (9.1)	1.05 (0.54 to 2.06)	16
Nguyen et al. [33]	Division of Gastrointestinal Surgical Oncology, Department of Surgery, Medical Center, Pittsburgh, PA, (USA)	Michels	P	29	0/30 (0)	0/30 (0)	0/30 (0)	0.77 (0.36 to 1.64)	19
Kim et al. [34]	Division of Pancreatobiliary Surgery, Department of Surgery, (Republic of Korea)	Unstandardized	P	11	2/15 (13.3)	0/15 (0)	0/15 (0)	–	20
Trofin et al. [35]	“Sf. Spiridon” County Clinical Emergency Hospital, Iasi (Romania)	Michels	Unspecified	28	4/45 (8.9)	2/45 (4.4)	2/45 (4.4)	–	19
Sanchez et al. [36]	Digestive Surgery Unit, Catholic University School of Medicine, Rome, (Italy)	Michels	P	25	0/25 (0)	0/25 (0)	0/25 (0)	–	17
Alexakis et al. [37]	Department of Surgery, Medical School, National and Kapodistrian University of Athens, Athens, (Greece)	Unstandardized	Unspecified	–	5/35 (14.2)	3/35 (8.6)	3/35 (8.6)	0.92 (0.69 to 1.22)	18
Crocetti et al. [38]	Pietro Valdoni Department of Surgery, Sapienza University of Rome, Rome, (Italy)	Unstandardized	P	–	0/44 (0)	0/44 (0)	0/44 (0)	1.00 (0.97 to 1.03)	19
Khang et al. [39]	Department of Surgery, Aga Khan University, Karachi, (Pakistan)	Hiatt	Unspecified	–	0/11 (0)	0/11 (0)	0/11 (0)	1.00 (0.88 to 1.14)	19
Wang et al. [40]	Department of Hepatobiliary and Pancreatic Surgery, The First Hospital of Jilin University, (China)	Michels/Hiatt	HPB	45	12/127 (9.4)	10/127 (7.9)	1/127 (0.8)	0.96 (0.69 to 1.33)	19
Giani et al. [41]	Division of Minimally–Invasive Surgical Oncology, Grande Ospedale Metropolitano Niguarda, Milan, Italy	Michels	P	13	0/14 (0)	0/14 (0)	0/14 (0)	1.01 (0.51 to 1.97)	20
Mangieri et al. [42]	Department of Surgery, Surgical Oncology Section, Wake Forest University Baptist Medical Center, NC (USA)	Unstandardized	P	–	0/36 (0)	0/36 (0)	0/36 (0)	0.73 (0.60 to 0.88)	18
Pyras et al. [3]	Department of Surgery, St. Josef Hospital, Ruhr University Bochum, Bochum, (Germany)	Hiatt	P	47	0/80 (0)	0/80 (0)	0/80 (0)	1.27 (0.89 to 1.81)	20

*RHA* Right hepatic artery; *CHA* Common hepatic artery; *CMT* Celiac-mesenteric trunk; *PDAC* Pancreatic ductal adenocarcinoma; *RR* Risk ratio; *HPB* Hepato-biliary pancreatic surgeon; *P* Pancreatic surgeon; *PDAC* Pancreatic ductal adenocarcinoma; *RR* Risk ratio; *MINORS* Methodological index for non-randomized studies

### Synthesis of results

#### Critical endpoints

**Fig. 2:**
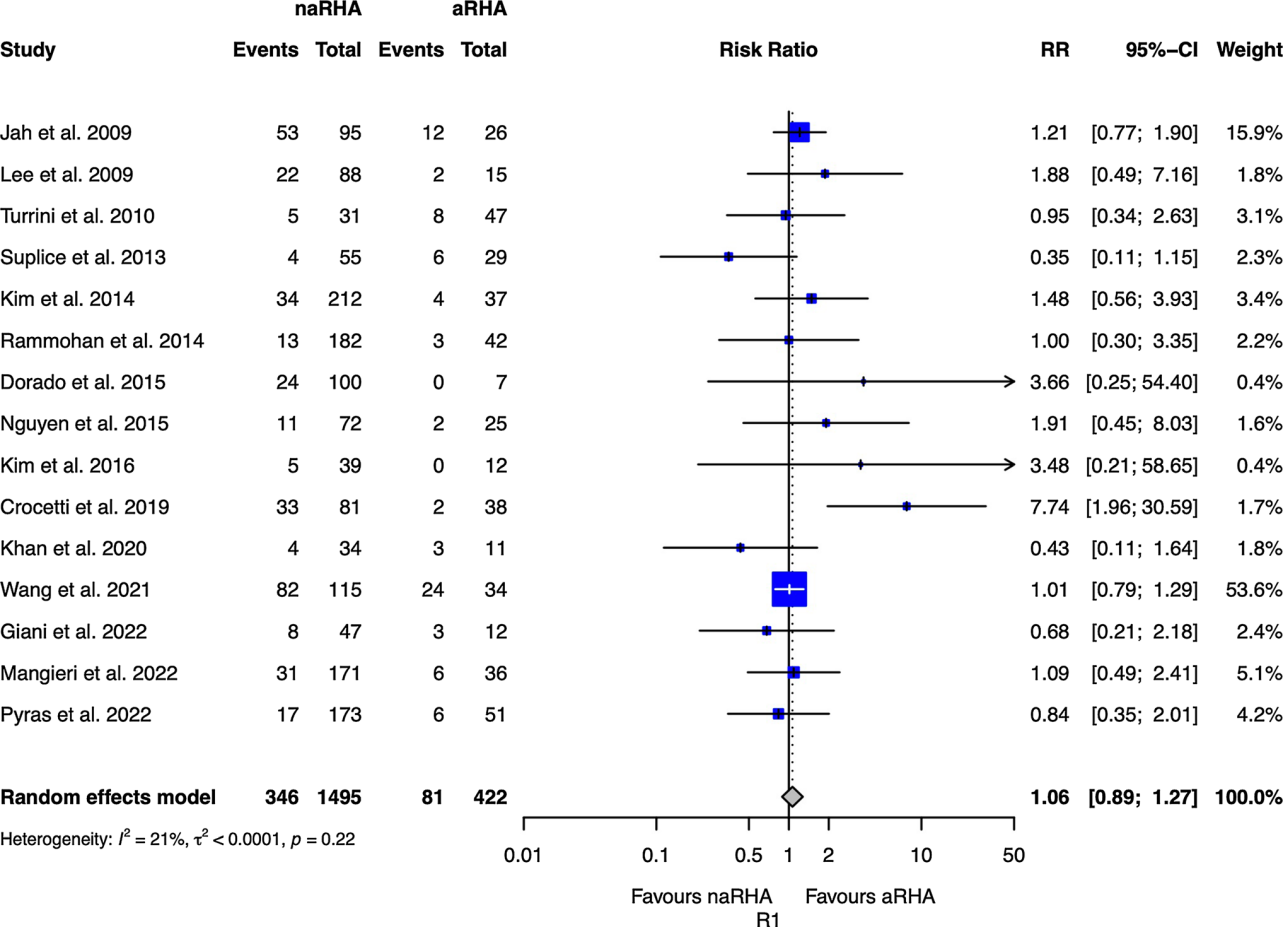
Forests plot of R1 resection rate in malignant tumors. *naRHA* Patients without aberrant right hepatic artery; *aRHA* Aberrant right hepatic artery; *RR* Risk ratio

**Fig. 3:**
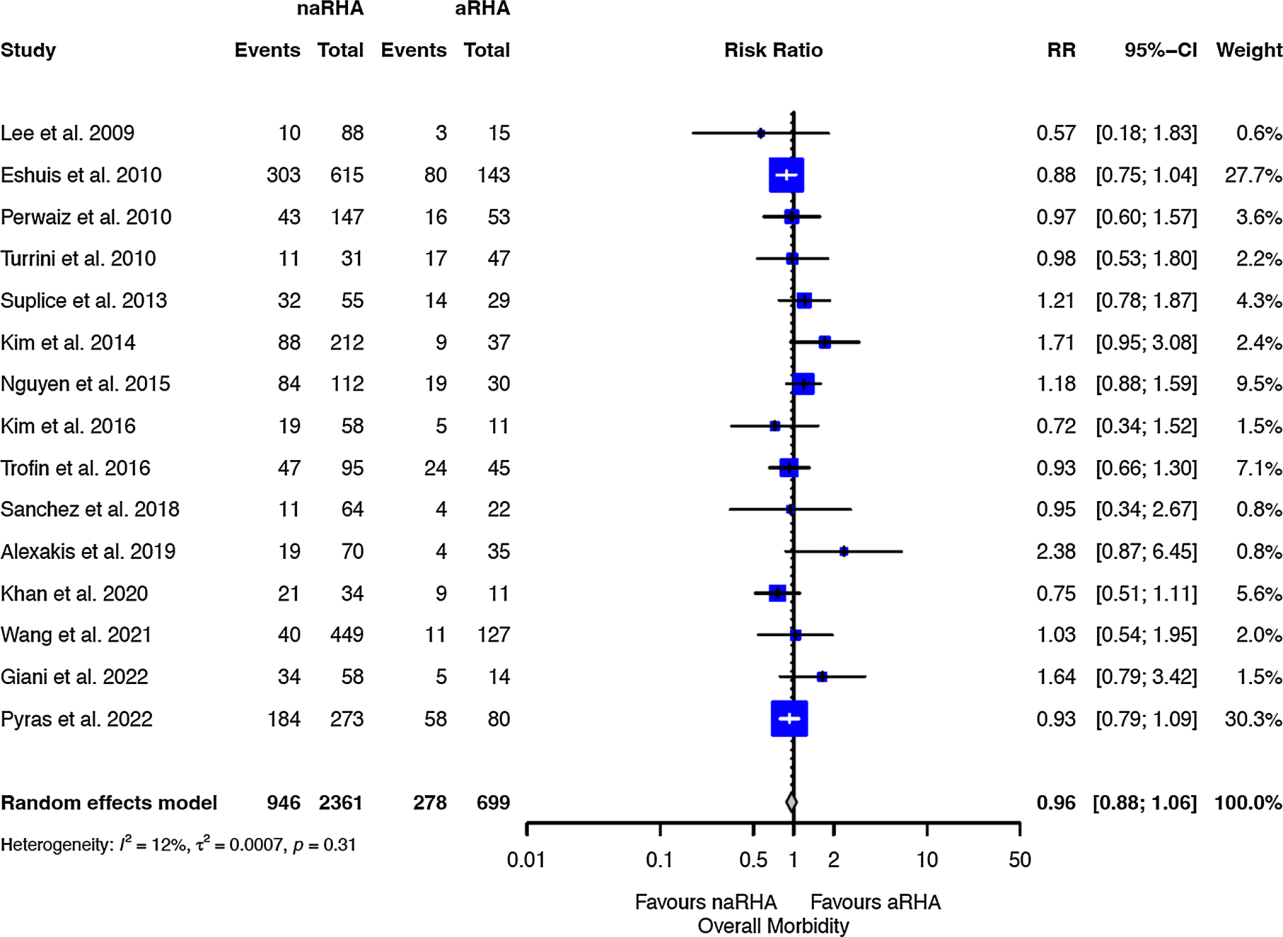
Forests plot of morbidity. *naRHA* Patients without aberrant right hepatic artery; a*RHA* Aberrant right hepatic artery; *RR* Risk ratio

**Fig. 4:**
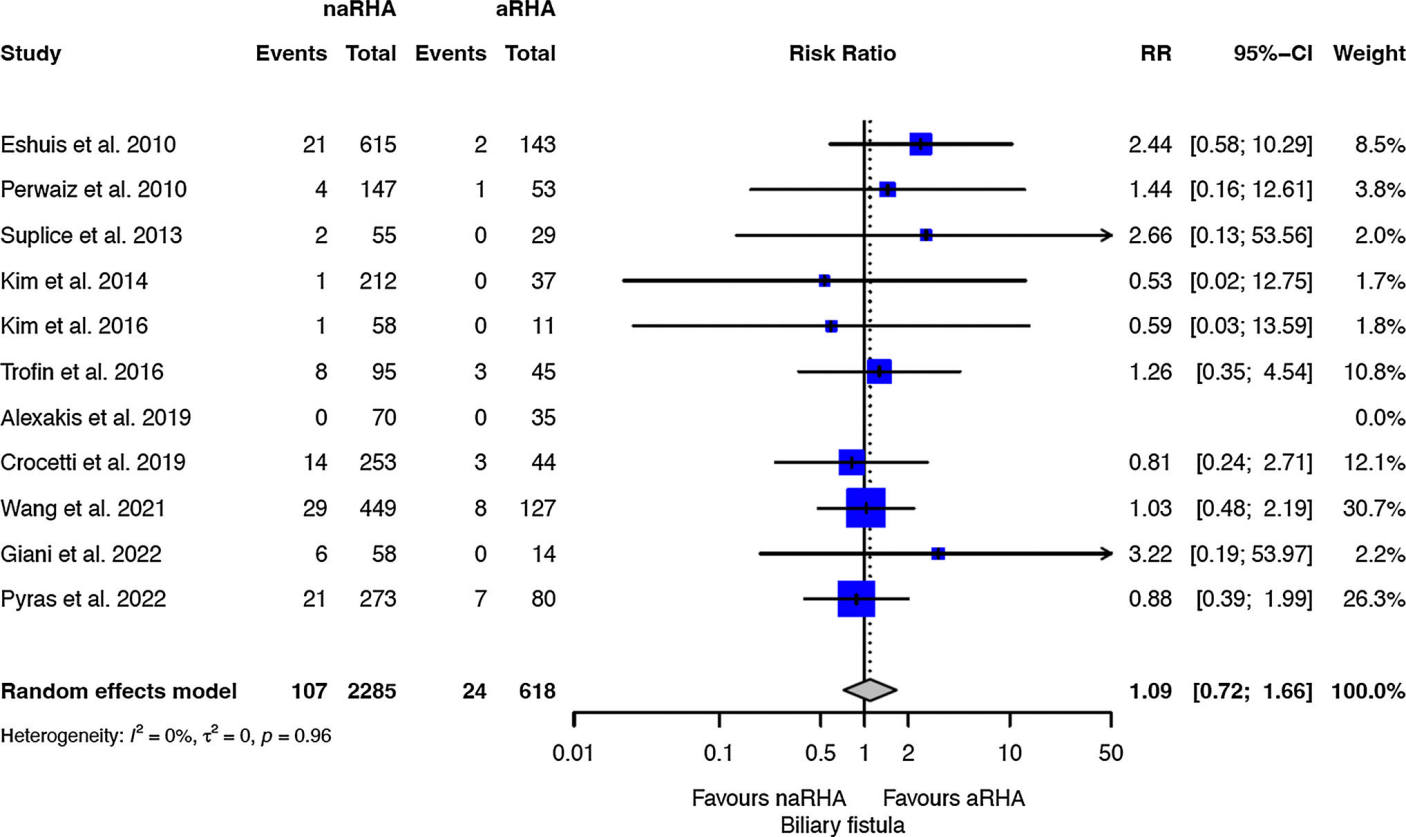
Forests plot of biliary fistula. *naRHA* Patients without aberrant right hepatic artery; *aRHA* Aberrant right hepatic artery

Table 2 shows the results for critical endpoints. The R1 rate was reported only in 16 studies, and no significant differences were observed between the two groups (Fig. 2): RR 1.06 (0.89 to 1.27, 95 CI). The OS was reported only in 8 studies, and the two groups have a similar risk of death (HR 0.95; 0.85 to 1.06, 95 CI). Postoperative morbidity (Fig. 3) and mortality were similar between the two groups, with a RR of 0.97 (0.88 to 1.06, 95 CI) and 0.81 (0.54 to 1.20, 95 CI), respectively. The biliary fistula rate was similar between the two groups (Fig. 4), with an RR of 1.09 (0.72 to 1.66, 95 CI). The RISs suggested that all equivalences are at risk for type II errors.

**Table 2:** Meta-analysis of all outcomes

Outcomes of interest	No. of studies	Event rate (%) or mean (SD)		RR/SMD/HR (95% CI)	*P*-value	ASS	RIS	C-Q, *I*^2^ (%)	*P*-value for reporting bias ^	
		na-RHA arm	a-RHA arm						Egger	Begg
*Critical endpoint*										
R1	15	346/1495 (23.1)	81/422 (19.1)	1.06 (0.89 to 1.27)	0.494	2,592	74,354	0.216; 21	0.429	0.102
OS	8	–	–	0.95 (0.85 to 1.06)	0.348	–	–	0.102; 41	0.052	1.000
Morbidity	15	946/2361 (40)	278/699 (39.7)	0.96 (0.88 to 1.06)	0.434	3,060	219,696	0.313; 12	0.172	0.586
Mortality	18	99/2903 (3.4)	31/814 (3.8)	0.81 (0.54 to 1.20)	0.289	3,614	20,117	0.989; 0	0.835	1.000
Biliary fistula	11	107/2285 (4.7)	24/618 (3.9)	1.09 (0.72 to 1.66)	0.681	2,501	43,025	0.960;0	0.329	0.531
*Secondary endpoints*										
CR-POPF	15	481/2774 (17.3)	121/734 (16.4)	1.10 (0.93 to 1.30)	0.249	3,508	15,688	0.985; 0	0.508	0.151
PPH	13	167/2555 (6.5)	43/676 (6.4)	1.00 (0.72 to 1.38)	0.989	3,231	26,248	0.998; 0	0.069	0.272
DGE	14	503/2716 (18.5)	147/723 (20.3)	0.84 (0.55 to 1.27)	0.407	3,439	25,904	0.003;58	0.081	0.352
LOS	11	16 ± 10	16 ± 9	0.80 (−0.70 to 2.38)	0.325	2,840	77,351	< 0.001;84	0.519	0.697
Operative time	13	323 ± 58	324 ± 61	−11.25 (−26.84 to 4.34)	0.157	3,059	26,807	< 0.001;95	0.086	0.272

*naRHA* Non-aberrant right hepatic artery; *aRHA* Aberrant right hepatic artery; *SD* Standard deviation; *RR* Risk ratio; *SMD* Mean difference; *HR* Hazard ratio; *C-Q* *P*-value of Cochran’s test; *I*^2^ Higgins test; (^) A reporting bias non-negligible is considered for *P* values < 0.10; *POPF* Clinical relevant postoperative pancreatic fistula; *PPH* Postpancreatectomy hemorrhage; *DGE* Delayed gastric emptying; *LOS* Length of stay; *OS* Overall survival; (–) Not applicable

#### Non-critical endpoints

Table 2 shows the results for non-critical endpoints. For CR-POPF, the RR was 1.10 (0.93 to 1.30, 95 CI) without significant differences between the two groups. Also, DGE and PPH have similar prevalence in the two groups, with an RR of 1.00 (0.72 to 1.38, 95 CI) and 0.84 (0.55 to 1.27, 95 CI). About LOS and operative time, no significant differences were found: MD of 0.80 (−0.70 to 2.38, 95 CI) and −11.25 (−26.84 to 4.34, 95 CI), respectively. The RISs suggested that all equivalences are at risk for type II errors.

#### Heterogeneity, meta-regression analysis, and publication bias

Concerning DGE, LOS, and operative time non-negligible heterogeneity was observed. None of the covariates explain the DGE and operative time heterogeneity. Meta-regression showed that MD is small in studies that adopted standardized classification (coefficient −3.53 ± 1.20; *P* = 0.003).

## Discussion

Our study demonstrated that the presence of a-RHA did not influence the short- and long-term outcomes of PD. The present meta-analytic cohort is the largest available and confirmed that a-RHA is frequent, and it can be found in one of five patients who underwent PD. This datum confirmed those reported by Hiatt for the general population [43]. Both types of a-RHA (relaced or accessory), by definition, arise from the SMA and pass on the back surface of the head of the pancreas, behind the portal vein, and through “meso-pancreas” to join in the hepatoduodenal ligament. The “meso-pancreas,” also called “retro-portal lamina” or “arterial margin,” was defined as the soft tissue between the superior mesenteric artery and the pancreatic parenchyma containing lymphatic, nervous, and vascular structures [44]. The R1 resection involving this margin was considered by several authors as the most important factor related to the local recurrences [45–48]. Based on these anatomical and oncological considerations, it seems logical to expect that the presence of a-RHA could increase the rate of R1 resection in the SMA, reducing overall survival. However, the present study suggested that the risk of R1 resection was similar with or without a-RHA. Nonetheless, the data should be read with caution for two main reasons. First, the majority of the studies did not distinguish the arterial margins from other pancreatic resection lines, such as posterior or anterior margins. Thus, the metanalytic R1 resection rate could be considered “dirty” data, including R1 resection margins not influenced by a-RHA presence. Secondly, the trial sequential approach suggested that the difference, between the two groups, is minimal, requiring a very large sample size to demonstrate or reject the null hypothesis. In other words, this large sample suggested that the oncological consequences of a-RHA, even if present, were so small to be indemonstrable. The results about OS survival confirmed this observation, even if this data is available only for 8 studies.

Another interesting observation is the effect of a-RHA on postoperative course. Damage or ligation of the a-RHA may lead to impaired perfusion and ischemia of the bile duct with a consecutive high risk of biliary leakage of the hepaticojejunostomy, liver abscess, or, rarely, hepatic failure [5]. However, our metanalytical cohort showed that the overall mortality, morbidity, and biliary fistula rate were similar without significant differences. Once again RIS values confirmed that the two anatomical conditions are so similar that confirming or rejecting the null hypothesis could require more than 10,000 patients. This observation did not surprise us, because the rate of a-RHA sacrificed or damaged is very small inferior to 10%. As expected, POPF, DGE, or PPH are similar in both groups. Indeed, it is well known and accepted that POPF depends on several factors such as pancreatic texture, Wirsung’s size, or BMI of patients [11]. The presence of an a-RHA did not influence the characteristics of the pancreas or the patients related to the POPF occurrence. Similarly, PPH and DGE are more related to POPF occurrence than damage or ligation of a-RHA. Operative time and LOS were similar between the two groups. However, the RISs were very large, suggesting that a large sample size was needed to reject or accept the null hypothesis. In other words, the differences are small and hard to demonstrate. LOS and operative time could be influenced by several factors, some of them related to the health care system or surgeon experience. It seems logical that the presence of a-RHA, per se, did not change the operative time or the duration of the postoperative course.

Strengths of the current study include the sample size and the methodology. This is the larger meta-analysis of the influence of a-RHA in patients who underwent PD. Moreover, trial sequential analysis eliminated the risk of false-negative or false-positive results. Limitations were several. First, the study included only retrospective comparative series. This design limited the intention-to-treat analysis. Indeed, when the sacrifice of a-RHA could be hypothesized by preoperative staging, neoadjuvant therapy should be considered [49]. However, only a few studies reported this data, and frequently, when a PD was not performed for a-RHA infiltration, the patients were not included in the series. For this reason, the data should be considered with caution. This metanalysis tells us about patients with head resectable pancreatic tumors without the clear involvement of arterial vessels, and the externalization of the results should be limited to this setting. On the contrary, when the tumor involves the a-RHA, neoadjuvant therapy should be performed, and resection with arterial reconstruction should be made without recurring inevitably total pancreatectomy [50].

A further limitation was that the paper included covered an extended time frame during which some changes in patient management were observed. The standardized definition of outcomes has changed during this period, such as the POPF definition or morbidity classification. Finally, it should be noted that some complications could also depend on the presence of a-LHA, even when pylorus-resecting PD was performed. Indeed, the ligation of the left gastric artery before the root of a-LHA could produce ischemia of the left hepatic lobe. Unfortunately, the included studies focused on a-RHA, and the role of a-LHA is impossible to evaluate in the present meta-analysis. Some limits could be ascribed to the methodology. TSA remains a retrospective method to analyze the trials sequentially. For this reason, TSA preserves the same risk of classical meta-analysis, namely a conclusion based on retrospective data-driven assumptions. Moreover, the TSA could result in a challenging and unusual methodology for clinicians.

In summary, the present meta-analysis confirms that the presence of a-RHA does not negatively affect the short-term and long-term clinical outcomes of PD. TSA confirmed that the differences between the two anatomical situations are so small that several hundreds of patients are required to definitively demonstrate the equivalence. Thus, additional studies about this topic could be useless. On the contrary, an interesting and new field of search could be the selective evaluation of arterial margins. This margin was rarely evaluated alone and in a standardized way in the available papers. Further prospective studies should be designed to evaluate if the presence of a-RHA increases the risk of R1 resection of arterial margins.
